# Phenotypic screening platform identifies statins as enhancers of immune cell-induced cancer cell death

**DOI:** 10.1186/s12885-023-10645-4

**Published:** 2023-02-17

**Authors:** Tove Selvin, Malin Berglund, Lena Lenhammar, Malin Jarvius, Peter Nygren, Mårten Fryknäs, Rolf Larsson, Claes R Andersson

**Affiliations:** 1 Department of Medical Sciences, Division of Cancer Pharmacology and Computational Medicine, Uppsala University, SE-75185 Uppsala, Sweden; 2 Department of Pharmaceutical Biosciences and Science for Life Laboratory, Uppsala University, Box 591, SE-751 24 Uppsala, Sweden; 3 Department of Immunology, Genetics and Pathology, Uppsala University, SE-75185 Rudbecklaboratoriet, Uppsala, Sweden

**Keywords:** Immuno-oncology, Drug screening, Repurposing, Small molecule drugs, Statins

## Abstract

**Background:**

High-throughput screening (HTS) of small molecule drug libraries has greatly facilitated the discovery of new cancer drugs. However, most phenotypic screening platforms used in the field of oncology are based solely on cancer cell populations and do not allow for the identification of immunomodulatory agents.

**Methods:**

We developed a phenotypic screening platform based on a miniaturized co-culture system with human colorectal cancer- and immune cells, providing a model that recapitulates part of the tumor immune microenvironment (TIME) complexity while simultaneously being compatible with a simple image-based readout. Using this platform, we screened 1,280 small molecule drugs, all approved by the Food and Drug Administration (FDA), and identified statins as enhancers of immune cell-induced cancer cell death.

**Results:**

The lipophilic statin pitavastatin had the most potent anti-cancer effect. Further analysis demonstrated that pitavastatin treatment induced a pro-inflammatory cytokine profile as well as an overall pro-inflammatory gene expression profile in our tumor-immune model.

**Conclusion:**

Our study provides an in vitro phenotypic screening approach for the identification of immunomodulatory agents and thus addresses a critical gap in the field of immuno-oncology. Our pilot screen identified statins, a drug family gaining increasing interest as repurposing candidates for cancer treatment, as enhancers of immune cell-induced cancer cell death. We speculate that the clinical benefits described for cancer patients receiving statins are not simply caused by a direct effect on the cancer cells but rather are dependent on the combined effect exerted on both cancer and immune cells.

**Supplementary Information:**

The online version contains supplementary material available at 10.1186/s12885-023-10645-4.

## Background

The many groundbreaking discoveries in molecular immunology and immuno-oncology over the last decades had a tremendous impact on the field of cancer treatment. Since the first immune checkpoint inhibitor (ICI), ipilimumab, was approved by the Food and Drug Administration (FDA) in 2011, immunotherapy has become a standard approach for cancer treatment alongside surgery, chemotherapy, and radiation [1]. However, despite clinical success for some cancer subtypes, limited response rates, safety issues, and complex pharmacokinetics remain major limitations. Immunomodulatory small molecule drugs have emerged as a promising complement to current immunotherapies as they may provide enhanced tissue penetration, improved bioavailability, and the ability to reach intracellular targets [2].

High-throughput screening (HTS) of small molecule drug libraries has greatly facilitated the discovery of new cancer drugs and has produced several FDA-approved chemotherapeutics that are currently used in the clinic [3]; however, most phenotypic HTS platforms used in the field of oncology are based solely on cancer cell populations and do not allow for the identification of immunomodulatory small molecules. To address this, Mo et al. recently developed a High-Throughput immunomodulator Phenotypic (HTiP) screening platform integrating cancer and immune cells [4]. In our study, using a similar approach, we utilized a miniaturized co-culture model system comprised of colorectal cancer (CRC) cells and immune cells to screen for small molecule drugs with immunomodulating activity.

Currently, ICI therapy for CRC is limited to a small subset of patients who harbor microsatellite instability-high (MSI-H) or deficient DNA mismatch repair (dMMR) tumors [5]. Furthermore, KRAS-driven cancers are notoriously difficult to treat and close to 50% of all CRC cases are characterized by mutated KRAS [6]. We established a model system comprised of the MSI-H and KRAS-mutated CRC cell line HCT116-GFP cultured as monoculture or co-cultured with peripheral blood mononuclear cells (PBMCs). Using this model, we screened 1,280 small molecule drugs, all approved by the FDA or other agencies, and identified the statin mevastatin as an enhancer of anti-cancer immunity. Upon evaluation of additional statins, the lipophilic statins mevastatin, simvastatin, pitavastatin, lovastatin and fluvastatin were all shown to potentiate activated PBMCs and increase CRC cell death. Furthermore, transcriptome-wide gene expression analysis of cancer and immune cells demonstrated an increased expression of tumor suppressor genes and an overall pro-inflammatory expression profile post-treatment with pitavastatin.

Statins are specific inhibitors of HMG-CoA reductase, the rate-limiting enzyme of the mevalonate pathway, widely prescribed to manage high cholesterol. Increasing evidence supports that the mevalonate pathway and its intermediate metabolites are critical for the growth and survival of certain cancers [7–9]. Concordantly, statins have been shown to reduce growth and induce apoptosis in various human cancer cell lines in vitro [8], [9]. Furthermore, several observational studies have reported that statin use is associated with decreased cancer-specific mortality in patients with a broad range of cancers [10]. Our findings added, we speculate that the clinical benefits described for cancer patients receiving statins [11–17] are not simply caused by a direct effect on the cancer cells but rather are dependent on the combined effect exerted on both cancer and immune cells. These data support the growing notion that statins are promising repurposing candidates for adjuvant cancer therapy.

## Methods

### Cell culture

HCT116-GFP, a human CRC cell line constitutively expressing green fluorescent protein (GFP), was obtained from AntiCancer Inc. (San Diego, CA, USA). Cell line authentication was performed by Eurofins Genomics (Ebersberg, Germany). Cells were cultured in McCoy’s 5 A medium supplemented with 10% heat-inactivated FBS, 2 mM L-glutamine, and Penicillin (100 U/mL)/ Streptomycin (100 µg/mL) (all from Sigma-Aldrich, St Louis, MO, USA). PBMCs from anonymous, healthy donors were isolated by Histopaque-1077 (Sigma) density gradient centrifugation and stored at -150 °C in FBS supplemented with 10% DMSO until used. All cells were cultured at 37 °C in 5% CO_2_.

### Characterization of PBMCs

Isolated PBMCs from three different donors were characterized using a CytoDiff flow cytometric system (Navios). Brifely, PBMCs were kept in PBS with 0.1% HSA and differential count of leukocytes was performed after staining with the following antibodies (all from BeckmanCoulter): anti-CD2-APC-AF750 (#B01681), anti-CD14-FITC (#B36297), anti-CD16-PC7 (#6,607,118), anti-CD19-PC5.5 (#B49211), anti-CD34-ECD (#B49202), anti-CD45-KO (#B36294), anti-CD6-PC7 (#A21692), and anti-CD294-PE (#A07413).

### Materials

The Prestwick Chemical Library containing 1,280 small molecule drugs (all approved by FDA or other agencies) dissolved in DMSO at 10mM, was purchased from Prestwick Chemical (France). Hit compounds used in validation experiments were purchased from Sigma-Aldrich or Selleckchem (Houston, TX, USA). The compounds were dissolved in DMSO or H_2_O and then diluted to desired final concentrations with culture medium. Mevastatin (Cat# M2537) and simvastatin (Cat# S6196) were purchased from Sigma-Aldrich. Pitavastatin (Cat# S1759) and remaining statins were purchased from Selleckchem. All statins were dissolved in DMSO and kept at a stock concentration of 10mM. Recombinant human IL-2 (Cat# 200-02) was purchased from Peprotech and anti-human CD3 was purchased from ThermoFisher (Cat# 16-0037-81, RRID: AB_468854).

### Drug screen

Monocultures were established by seeding HCT116-GFP cells (1,000 cells/50µL/well) in 384-well Nunc plates (ThermoFisher Scientific). After 24 h preculturing, PBMCs were added at a 1:1 ratio (1,000 cells/50µL/well) to establish co-cultures. At the same time as PBMCs were added, the medium of the co-cultures was supplemented with anti-CD3 (final concentration 100 ng/mL) and IL2 (final concentration 10 ng/mL). The Prestwick drug library was then added to both mono- and co-cultures at a final concentration of 10 µM using an Echo Liquid Handler 550 (Labcyte). The treated plates were placed in the Live-Cell Analysis System IncuCyte S3 (Essen Bioscience, Ann Arbor, MI, USA) and incubated with drugs for 96 h. The cancer cell viability was indirectly monitored by continuously measuring GFP expression every 4 h during the treatment.

### Assay quality assessment

Z-factors, estimated from sample means and standard deviations (SD) of positive and negative controls, are commonly calculated to assess the quality of assays used for screening; a Z-factor of 1 is ideal, between 0.5 and 1 is an excellent assay, between 0 and 0.5 is a marginal assay, and a negative Z-factor means that the signal from positive and negative controls could overlap [18]. Here, SD and mean values were calculated based on GFP intensity in cell cultures without added drugs. Z-immunomodulation (Z_M_) and Z-immunopotentiation (Z_P_) were defined as:

$${\text{Z}}_{\text{M}}=1-\frac{3\left({\text{S}\text{D}}_{\text{P}\text{B}\text{M}\text{C}}+{\text{S}\text{D}}_{\text{H}\text{C}\text{T}116}\right)}{{| \text{m}\text{e}\text{a}\text{n}}_{\text{P}\text{B}\text{M}\text{C}}-{\text{m}\text{e}\text{a}\text{n} }_{\text{H}\text{C}\text{T}116} |}$$

and

$${\text{Z}}_{\text{P}}=1-\frac{3\left({\text{S}\text{D}}_{\text{P}\text{B}\text{M}\text{C}}+{\text{S}\text{D}}_{Co-culture}\right)}{{| \text{m}\text{e}\text{a}\text{n}}_{\text{P}\text{B}\text{M}\text{C}}-{\text{m}\text{e}\text{a}\text{n} }_{\text{c}\text{o}-\text{c}\text{u}\text{l}\text{t}\text{u}\text{r}\text{e}} |}$$

### Selection of hit compounds

According to the Bliss Independence Model [19], the product of the reduced cell viability (% of control) induced by two single drugs with independent effects is expected to be equal to the viability of the combination of the two drugs. Defining a Bliss score as B = Viability Drug 1 ⋅ Viability Drug 2 − Viability Drug 1 + 2 = 0, a positive Bliss score thus indicates that the viability of the combination is lower than expected if effects are independent and therefore indicates synergy. Here, the Bliss model was applied to detect small molecule drugs synergizing with anti-CD3/IL2 activated PBMCs. A Bliss score was calculated for each compound and used to rank them based on immune cell potentiation.

### Validation experiments

HCT116-GFP cells were seeded in 384-well Nunc plates (1,000 cells/50µL/well) or 96-well Nunc plates (5,000 cells/100µL/well) and grown as monoculture or co-cultured with anti-CD3/IL2 activated PBMCs at a 1:4 ratio. Newly purchased and freshly prepared hit compounds and statins were added at desired concentrations to 384-well plates using an Echo Liquid Handler 550 and manually to 96-well plates. Viability was indirectly measured by quantification of GFP using an IncuCyte S3.

### Measurement of cytokines

HCT116-GFP cells (5,000 cells/100µL/well) were seeded in 96-well Nunc plates and co-cultured with anti-CD3/IL2 activated PBMCs. Co-cultures were treated with DMSO vehicle (0.01%, 0.1%), dexamethasone (DEX) (10 µM), mevastatin (1 µM, 10 µM), simvastatin (1 µM, 10 µM), or pitavastatin (1 µM, 10 µM) for 24 h before supernatants were collected. Cytokine levels in the supernatants were measured using a Bio-Plex Pro Human Cytokine 5-plex assay (Bio-Rad, USA) according to the manufacturer’s instructions. Briefly, the cytokines of interest are bound to magnetic beads via antibodies and subsequently detected using biotinylated antibodies with a fluorescent reporter. Fluorescence was measured using a Luminex MAGPIX system (Bio-Rad, USA) and concentrations were calculated using standard curves.

### RNA microarray

On day 0, HCT116-GFP (1,5*10^5^ cells/3mL/well) were seeded in 6-well Nunc plates. On day 1, PBMCs (6*10^5^ cells/3mL/well) stimulated with anti-CD3 (final concentration 100 ng/mL) and IL2 (final concentration 10 ng/mL) were seeded as monocultures or added to cancer cells precultured for 24 h at a 1:4 ratio. HCT116-GFP monocultures, PBMC monocultures, and co-cultures were then treated with DMSO vehicle (0.1%) or pitavastatin (1 µM, 10 µM) for 24 h before RNA was isolated using a RNeasy Mini Kit (Cat# 74,104, Qiagen, CA, USA) according to the procedure Purification of total RNA from animal cells using spin technology. RNA quantity was measured using a NanoDrop One spectrophotometer (Thermo Scientific). RNA quality assessment using an Agilent 2100 Bioanalyzer system and gene expression analysis using a Human Clariom S array were performed by the Array and Analysis Facility, Department of Medical Sciences, Uppsala University. Data normalization and analysis were performed using the Transcriptome Analysis Console, version 4.0.2.15, provided by Thermo Fisher. The raw data was normalized separately for PBMC, HCT116-GFP and co-culture using default settings.

## Results

### Phenotypic screening platform identifies mevastatin as an enhancer of immune cell-induced cancer cell death

**Fig. 1:**
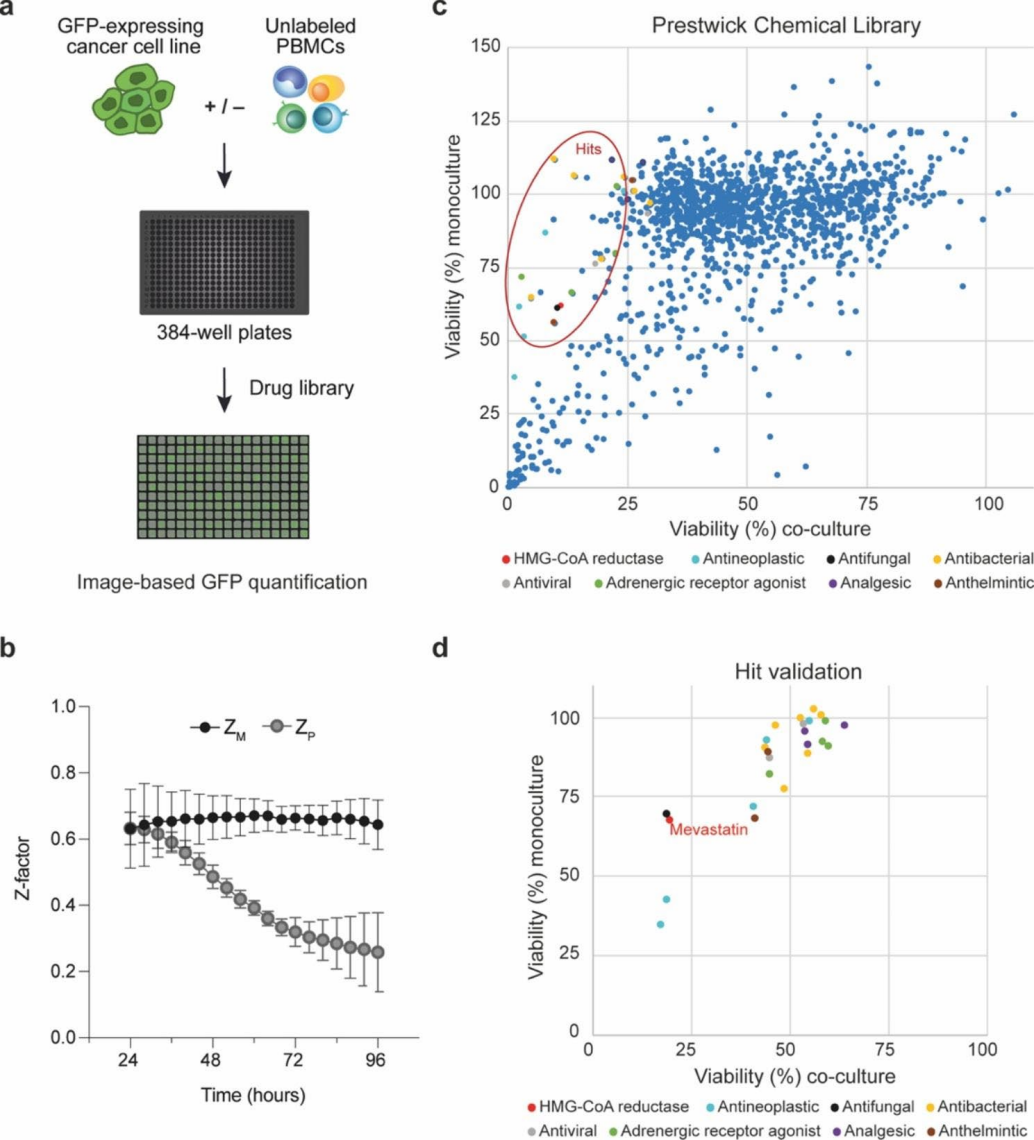
**Immuno-oncology screening platform identifies statins as enhancers of immune cell-induced cancer cell death. (a)** Schematic of the miniaturized 384-well plate-based model system used for drug screening: HCT116-GFP cells cultured as monoculture or co-cultured with anti-CD3/IL2 activated PBMCs at a 1:1 ratio. **(b)** Z-immunomodulation (Z_m_) and Z-immunopotentiation (Z_p_) calculated over time, based on GFP-signal in untreated HCT116-GFP cells in monoculture, PBMCs in monoculture, or co-cultured cells quantified every 4 h for 96 h. Results are shown as mean ± SD from four independent experiments (n = 64). **(c)** Viability of HCT116-GFP cells, indirectly measured by image-based quantification of GFP intensity, in monoculture (y-axis) and in co-culture (x-axis) 72 h after addition of the Prestwick Chemical Library drugs at a final drug concentration of 10 µM. Hit compounds selected for validation experiments are encircled in red. **(d)** Viability of HCT116-GFP cells, cultured as monoculture or co-cultured with PBMCs at a 1:4 ratio, 72 h after treatment with newly purchased hit compounds at a final concentration of 10 µM for validation (n = 3)

To detect small molecule drugs with immunomodulating activity in vitro, we developed an immuno-oncology screening platform. The human CRC cell line HCT116-GFP was cultured as monoculture or co-cultured with anti-CD3/IL2 activated PBMCs at a 1:1 ratio in a 384-well plate format (Fig. 1a). To assess the quality of the assay, two Z-factors were calculated; Z_M_, reflecting the dynamic range of the assay, and Z_P_, reflecting the window for detecting treatment-induced immunopotentiation (Supplementary Figure S1a). The dynamic range of the assay remained excellent over time, demonstrated by Z_M_ ≥ 0.5 for all time points, while Z_P_ decreased over time (Fig. 1b). At 72 h, both Z_M_ and Z_P_ indicated a robust assay with low variation across four independent experiments, hence 72 h was the selected time point for evaluation of the compound library.

The Prestwick Chemical Library was screened in mono- and co-culture. Viability, expressed as surviving cells relative to untreated controls, was indirectly measured by image-based quantification of GFP intensity (example images, see Supplementary Figure S1b). The Bliss Independence Model was then applied to identify compounds that synergized with activated PBMCs to reduce the viability of HCT116-GFP cells. A Bliss score was calculated for each compound in the drug library and 25 hit compounds were selected based on this score and literature (Fig. 1c and Supplementary data 1). Hit validation was performed using newly purchased and freshly prepared compounds and a cancer cell: PBMC ratio of 1:4. Most hit compounds were less efficient in the validation experiments than in the screen. However, the HMG-CoA reductase mevastatin, together with a few additional compounds, demonstrated a reproducible immunopotentiating effect (Fig. 1d).

### Lipophilic statins potentiate pro-inflammatory responses in vitro

**Fig. 2:**
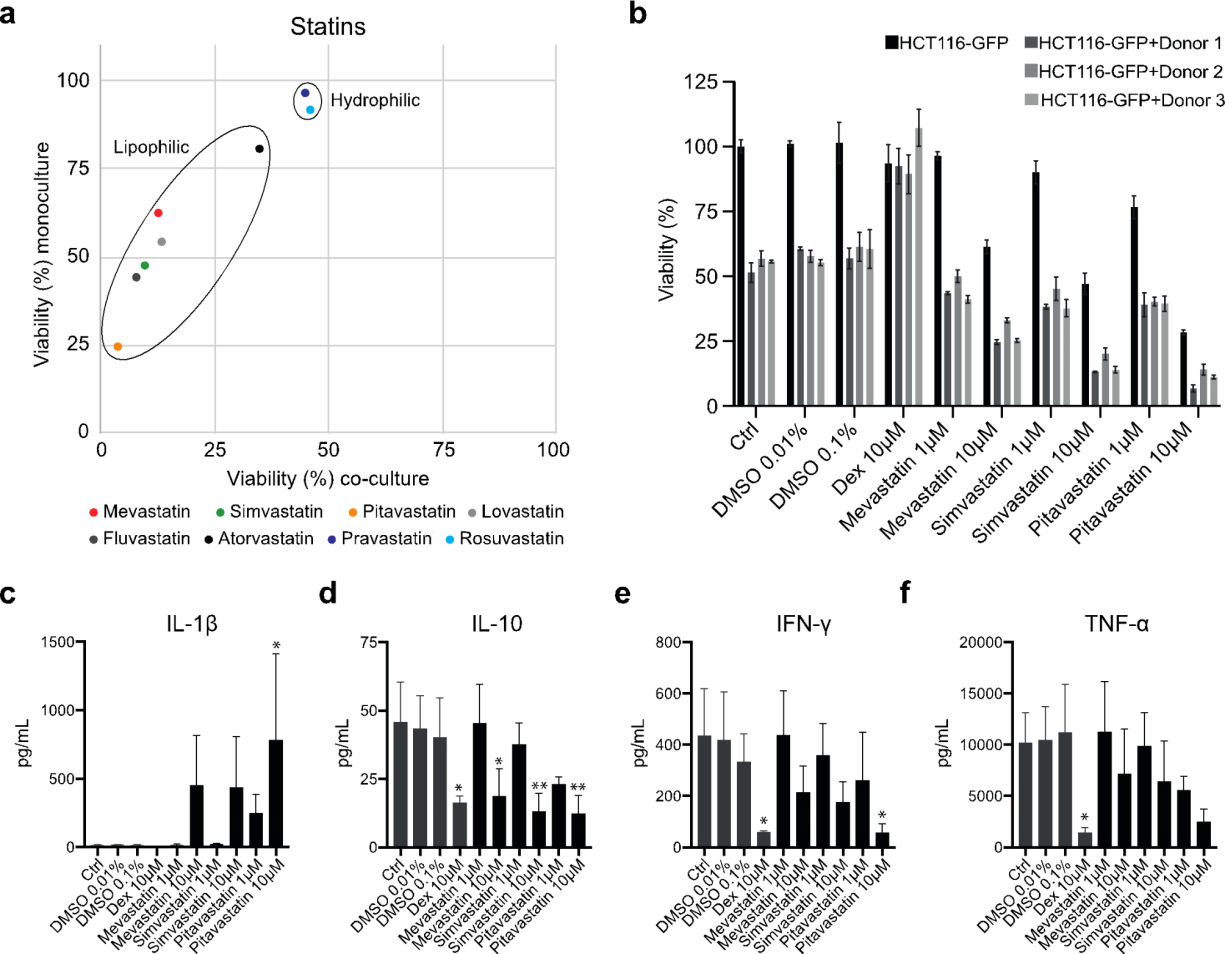
**Lipophilic statins potentiate pro-inflammatory responses in vitro. (a)** Viability of HCT116-GFP cells in mono- and co-cultures 72 h after treatment with statins at a final concentration of 10 µM (n = 3). **(b)** Viability measured in HCT116-GFP cells in monoculture or co-cultures with PBMCs from three different donors, treated with DMSO vehicle (0.01%, 0.1%), dexamethasone (DEX) (10 µM), or one of the three statins mevastatin, simvastatin, and pitavastatin (1 µM, 10 µM) for 72 h. Data is shown as mean ± SEM (n = 3). Luminex MAGPIX system assessment of **(c)** IL-1β, **(d)** IL-10, **(e)** IFN-γ, and **(f)** TNF-α concentrations measured in co-culture supernatants collected after 24 h treatment. Data shown as mean ± SD from three independent experiments. One-way Anova with Dunnett’s multiple comparison test, compared to DMSO vehicle, *adjusted P-value < 0.05, **adjusted P-value < 0.01

When analyzing additional members of the statin drug family, the lipophilic statins mevastatin, simvastatin, pitavastatin, lovastatin, and fluvastatin were all shown to increase PBMC-induced killing of HCT116-GFP cells (Fig. 2a). Mevastatin, simvastatin, and pitavastatin were selected for further evaluation. HCT116-GFP cells were grown as monoculture or co-cultured with activated PBMCs from three different donors which were characterized using a CytoDiff flow cytometric system (Supplementary Figure S2). Mono- and co-cultures were treated with DMSO vehicle (0.01%, 0.1%), DEX (10 µM), or one of the three statins (1 µM, 10 µM) for 72 h (Fig. 2b). Activated PBMCs alone decreased the viability of the HCT116-GFP cells to an average of 55% (51%, 57%, and 56%, respectively for each donor). This immune-mediated decrease in cancer cell viability was completely inhibited by treatment with the anti-inflammatory corticosteroid DEX. Treatment with statins on the other hand reduced the viability of HCT116-GFP cells in co-culture. The most potent anti-cancer effect was observed for pitavastatin both in mono- and co-culture. In monoculture, treatment with 10 µM pitavastatin reduced the viability of HCT116-GFP cells to 28%. In the presence of PBMCs the viability was, on average across the three donors, reduced to 11% (Fig. 2b). Moreover, although a greater reduction in cancer cell viability was observed at 10 µM, measuring viability over time demonstrated that 1 µM statin treatment was sufficient to enhance anti-cancer immunity. At 1 µM, mevastatin and simvastatin had little to no direct effect on HCT116-GFP cells in monoculture, yet a clear decrease in cancer cell viability was observed in treated compared to untreated co-cultures over time (Supplementary Figure S4).

Next, a Luminex MAGPIX system was used to measure cytokine concentrations in co-culture supernatants after 24 h treatment. In accordance with the literature [20], [21], we observed an apparent increase in secreted IL-1β following statin treatment (Fig. 2c). Furthermore, all three statins induced a dose-dependent decrease in IL-10, IFN-γ, and TNF-α concentrations (Fig. 2d-f). Statin-induced inhibition of the immunosuppressive cytokine IL-10 has been described previously, for example in a study by Perucha et al. that also demonstrated the specificity of this effect as it could be prevented by the addition of mevalonate [22]. These data, generated by us and others, argue against the general perception that pleiotropic effects of statins are solely immunosuppressive.

### Pitavastatin treatment increases the expression of tumor suppressor genes

**Fig. 3:**
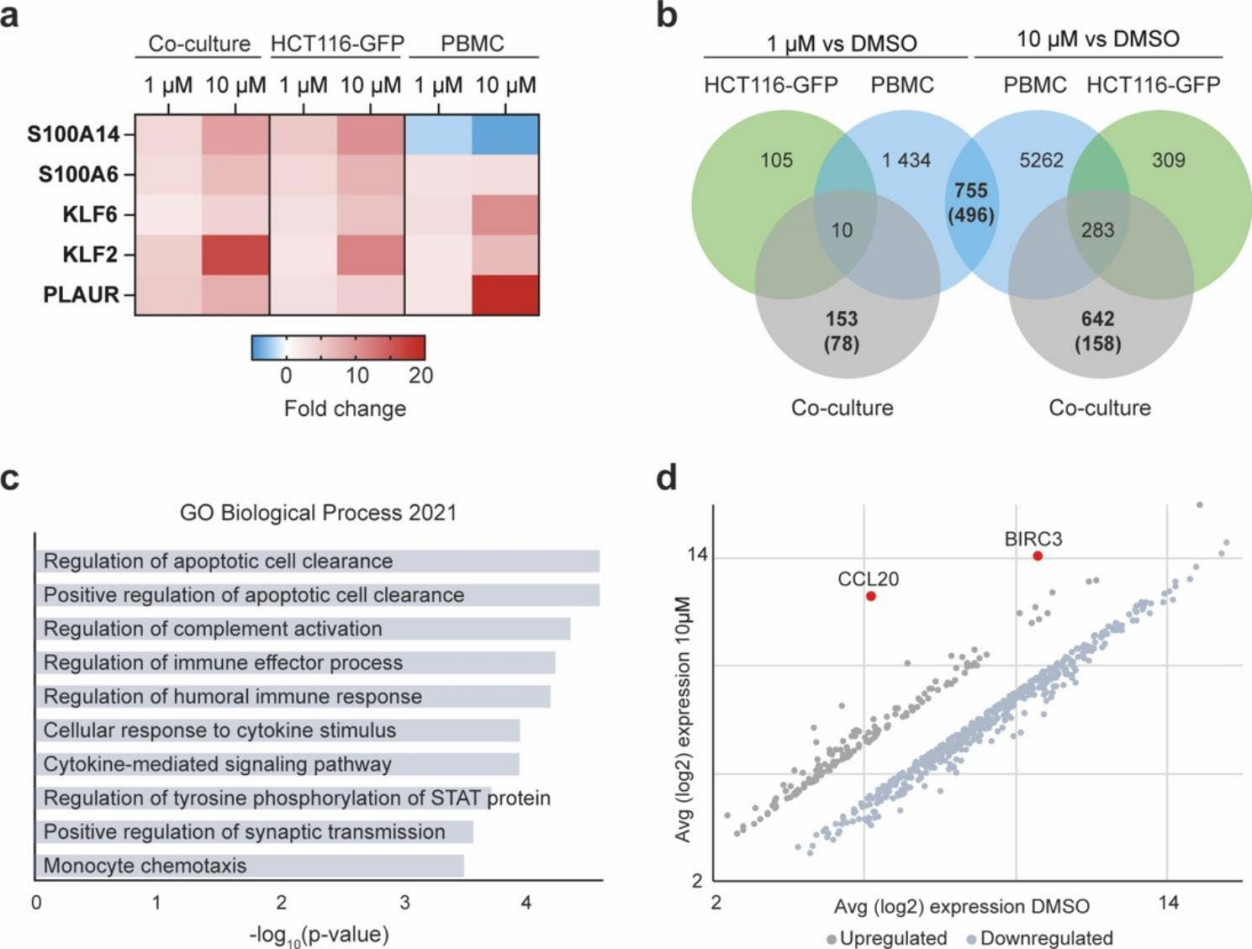
**Pitavastatin treatment increases the expression of tumor suppressor genes and induces an overall pro-inflammatory gene expression profile in vitro. (a)** Fold change compared to DMSO vehicle for DEGs (fold change > 2) common to all cultures (HCT116-GFP monoculture, PBMC-monoculture, and co-culture) after 24 h treatment with pitavastatin (1 µM or 10 µM). **(b)** Venn diagram showing the number of DEGs (fold change > 2), and for selected cultures the number of upregulated DEGs within brackets, 24 h post treatment with pitavastatin (1µM or 10µM) compared to DMSO vehicle. **(c)** Enriched terms (adjusted P-value < 0.05) obtained from enrichment analysis using the GO Biological Process 2021 database. The enrichment analysis was performed using the 158 DEGs (fold change > 2) that were uniquely upregulated in co-culture treated with 10 µM pitavastatin. **(d)** Average log2 expression of DEGs found in co-culture treated with 10 µM pitavastatin but not in either of the monocultures

Studying gene expression profiles in response to treatment is a well-established approach for investigating a compound’s mode of action [23], [24]. Herein, we performed transcriptome-wide gene expression profiling on HCT116-GFP cells and PBMCs. Monocultured and co-cultured cells were treated with DMSO vehicle or pitavastatin (1 µM, 10 µM) for 24 h before gene expression was analyzed using a Human Clariom S array. Reduced cholesterol levels following statin treatment trigger upregulation of the transcription factor sterol regulatory element-binding transcription factor 2 (SREBF2) and, consequently, increased expression of SREBF2-target genes involved in cholesterol synthesis and degradation pathways [10], [25]. As expected, we observed an apparent upregulation of *SREBF2* and several downstream target genes in HCT116-GFP cells in monoculture and in co-culture post pitavastatin treatment (Supplementary data 2). In PBMCs however, *SREBF2* was not found among differentially expressed genes (DEGs). Apart from genes directly involved in the mevalonate cascade, only five genes were found to be differentially expressed (fold change > 2) in all cultures (HCT116-GFP monoculture, PBMC monoculture, and co-culture) treated with pitavastatin (1µM and 10µM) compared to DMSO vehicle (Fig. 3a). Two of these genes, *S100A6* and *S100A14*, encodes calcium-binding S100 proteins. *S100A14*, which has been shown to exert tumor-suppressive effects in gastrointestinal cancers [26], was upregulated in a dose-dependent manner in HCT116-GFP cells while a dose-dependent decrease was observed in PBMCs. Two additional genes acting as tumor suppressors, krüppel-like factor 2 (*KLF2*) and 6 (*KLF6*), were found among the 5 DEGs common to all pitavastatin treated cultures. Expression of *KLF2* and *KLF6* has previously been shown to increase in cancer cells following treatment with the lipophilic statin lovastatin and an association between induction of these genes and apoptosis has been demonstrated [27].

### Pitavastatin induces an overall pro-inflammatory gene expression profile

Contrary to the general perception of statins as anti-inflammatory, a study by Xia et al. suggests that lipophilic statins are potent vaccine adjuvants exerting their immune-regulatory function by preventing protein prenylation and, thereby, increasing antigen presentation and T cell activation [28]. In agreement with this, we observed what appears to be an overall stimulatory effect on T cell-mediated immunity. When evaluating treatment-induced gene expression profiles in PBMC-monocultures, we identified 496 common upregulated DEGs (fold change > 2) in PBMC-monocultures treated with 1 µM and 10 µM pitavastatin (Fig. 3B, Supplementary data 3). Enrichment analysis of these upregulated DEGs, using the GO Biological Process 2021 database, identified biological processes such as antigen processing and presentation, positive regulation of leukocyte cell-cell adhesion, regulation of CD8^+^ alpha-beta T cell proliferation, and positive regulation of T cell-mediated immunity among the top enriched terms (Supplementary data 3). Next, we analyzed genes that were differentially expressed in the co-culture but not in either of the monocultures (Fig. 3b). After 24 h treatment with pitavastatin (10 µM), 158 DEGs (fold change > 2) (Supplementary data 4) were uniquely upregulated in co-culture. Enrichment analysis of these genes identified processes such as positive regulation of apoptotic cell clearance, regulation of immune effector processes, and monocyte chemotaxis among the top 10 enriched terms (adjusted P-value < 0.05) (Fig. 3c). Furthermore, two inflammatory mediators, *CCL20* and *BIRC3*, stood out in co-cultures treated with 1µM (Supplementary Fig. S4) and with 10 µM pitavastatin (Fig. 3d) as the two genes with the highest increases in expression fold change. Interestingly, when analyzing the transcriptome of epithelial cells, *CCL20* is typically found among the most highly induced genes following pro-inflammatory stimuli [29], suggesting a pro-inflammatory effect of pitavastatin treatment.

## Discussion

Over the past decade, advances in the development of in vitro and ex vivo model systems have contributed to an increased understanding of anti-cancer immunity. However, model systems that recapitulate the TIME and simultaneously are compatible with high-throughput phenotypic drug screening are still scarce. We developed a phenotypic screening platform comprised of GFP-labeled human CRC cells and human PBMCs. Utilizing GFP-labeled cancer cells grown both as monoculture and co-cultured with a mixture of immune cells provides a model that recapitulates at least part of the complexity of the TIME and simultaneously enables a simple image-based readout. However, GFP-labeling of cancer cells has been described to increase the immunogenicity of the cancer cells [30], which should be taken into consideration when designing an immuno-oncology model system with GFP-labeled target cells. In our model system, co-culturing HCT116-GFP with activated PBMCs reduced the viability of HCT116-GFP cells to an average of 50% (supplementary data 1), leaving a sufficient window for identification of immunomodulators. Using this approach, the statin mevastatin was identified as one of few small molecule drugs in the Prestwick chemical library with the ability to potently and reproducibly enhance immune cell-induced cancer cell death (Fig. 1d).

A considerable amount of data from preclinical and clinical studies demonstrate that statins have tumor-suppressing properties [31]. Furthermore, statin treatment has been associated with lower cancer risk [11], [12] and with increased survival of cancer patients [11], [13]. In our study, we observed a moderate direct effect on HCT116-GFP cell viability in monoculture. However, a potent anti-cancer effect was observed when immune cells were present during statin treatment (Fig. 2b, Supplementary Figure S4). Statins are generally considered anti-inflammatory [32]. The literature is however conflicted as several members of the statin drug family have been shown to exert various pro-inflammatory effects. For example, statin-induced decrease in protein prenylation has been shown to increase secretion of the pro-inflammatory cytokine IL-1β which plays a key role during the initiation of inflammatory responses [20], [21]. Additionally, statin treatment has been described to inhibit the secretion of anti-inflammatory IL-10 [22]. In accordance with the literature, we observed an increase in secreted IL-1β and decreased IL-10 in co-cultures after 24 h statin-treatment (Fig. 2c and d). We also observed a dose-dependent decrease in secreted TNF-α (Fig. 2e). Blocking TNF-α has emerged as a possible strategy to improve anti-cancer responses to ICI treatment as it is thought to enhance specific immune responses by limiting activation-induced cell death of cytotoxic T cells [33], [34]. Interestingly, recent studies suggest that statin treatment is associated with improved clinical outcomes for patients receiving therapy with ICIs [14–17].

As pitavastatin demonstrated the most potent anti-cancer effect in both monoculture and co-culture (Fig. 2b), and had the greatest impact on cytokine secretion (Fig. 2c-f), pitavastatin was selected for further analysis. Performing transcriptome-wide gene expression analysis demonstrated that pitavastatin treatment increased the expression of tumor suppressor genes as well as induced an overall pro-inflammatory expression profile in our tumor-immune model in vitro (Fig. 3 and Supplementary data 3). Interestingly, *BIRC3* and *CCL20* were the most highly induced genes following pitavastatin treatment in co-culture. *BIRC3* encodes one of the eight members of the inhibitor of apoptosis protein (IAP) family, a protein family that has been extensively investigated for its involvement in the evasion of apoptosis in tumors. Although IAPs are collectively referred to as pro-oncogenic, the literature regarding *BIRC3* (cIAP2) is conflicted; multiple clinical studies have demonstrated an unfavorable contribution of *BIRC3* inactivation in cancer patients [35]. Furthermore, contrary to what was previously believed, IAPs are not solely inhibitors of apoptosis but also major positive regulators of innate immunity and inflammatory responses [36]. *CCL20* is also an important mediator of immunity, especially in the intestines as it contributes to the development of intestinal lymphoid tissues, structures that facilitate adaptive immune responses in the intestine [29].

We speculate that the mechanisms through which pitavastatin enhances anti-tumor immunity are mediated by a combined effect on cancer and immune cells. In a study published by Nam et al. in 2021 simvastatin treatment was shown to inhibit KRAS prenylation, resulting in induction of immunogenic cell death (ICD) in KRAS mutant CRC cells [37]. Mutated KRAS, commonly referred to as an “undruggable target”, is a driver of various cancers and is associated with suppressed anti-cancer immunity [38]. Nam et al. suggest that mutated KRAS could be a molecular target for statins and provides encouraging in vivo data from syngeneic tumor models. The ability to induce ICD in KRAS mutant tumors could explain the potentiation of anti-tumor immunity in our model system, although further investigation as to whether pitavastatin shares this mechanism is required. Moreover, 24 h treatment with pitavastatin was enough to induce an overall pro-inflammatory gene expression profile in PBMC monocultures (Supplementary Data 3), indicating that pitavastatin may exert additional mechanisms that directly targets the immune cells. The characterization of PBMCs obtained from the different donors demonstrated that Donor 1 had a higher percentage of monocytes compared to Donor 2 and 3 (Supplementary Figure S2). Statin treatment of co-cultures established with immune cells from Donor 1 consistently generated a slightly greater reduction of cancer cell viability. Taken together with a significant increase in secreted IL-1β following pitavastatin treatment (Fig. 2c), we speculate that monocytes could be a subpopulation driving the immuno-oncological effect of pitavastatin.

Important for putting our findings in a clinical context, statins are rapidly absorbed in humans following administration and mean concentrations in human serum are in the low nM range. In contrast, most pleiotropic effects of statins, when evaluated in vitro or in rodent models, are observed at concentrations in the µM range [39]. Statin treatment is, however, usually administered continuously, making the exposure time in humans substantially longer than in experimental settings. Additionally, the bioavailability of the synthetic statin pitavastatin is considerably higher than that of other statins. Unlike many other statins, pitavastatin is not metabolized by the enzyme CYP3A4 and the low level of metabolization by CYP2C9 is clinically insignificant, resulting in increased duration of action and decreased probability of drug-drug interactions [40]. Furthermore, while cellular uptake of hydrophilic statins requires active carrier-mediated transport through transporters that are mainly expressed on hepatocytes, cellular uptake of lipophilic statins such as pitavastatin occur through passive diffusion. Due to efficient first-pass uptake, all statins are selective for effect in the liver; lipophilic stains can however also diffuse across cell membranes in extrahepatic tissue [41]. This discrepancy in uptake mechanism between the two groups of statins is likely part of the explanation as to why lipophilic but not hydrophilic statins were shown to enhance anti-tumor immunity in the present study.

Difficulties in administrating statins at high concentrations make it unlikely that statins will be used as a monotherapy in the field of oncology. However, our data suggest that pitavastatin not only exerts a direct effect on cancer cells but also enhances anti-cancer immunity in vitro. The unique metabolic profile of pitavastatin, taken together with a good safety profile and increasing observations of anti-cancer effects, warrants further investigation of pitavastatin as a repurposing candidate for cancer therapy. An interesting next step would be to investigate if pitavastatin may enhance the effect of ICIs and, thus, could be used as an adjunct in such therapy.

## Conclusion

Our study provides an in vitro phenotypic screening approach for the identification of immunomodulatory agents and thus addresses a critical gap in the field of immuno-oncology. Furthermore, our pilot screen identified statins, a drug family gaining increasing interest as repurposing candidates for cancer treatment, as enhancers of immune cell-induced cancer cell death.

## Electronic supplementary material

Below is the link to the electronic supplementary material.

Supplementary Material 1

Supplementary Material 2

Supplementary Material 3

Supplementary Material 4

Supplementary Material 5

## Data Availability

The microarray data generated in this study has been deposited in NCBI’s Gene Expression Omnibus (GEO) database (RRID:SCR_005012) and is accessible through GEO Series accession number GSE207280 (https://www.ncbi.nlm.nih.gov/geo/query/acc.cgi?acc=GSE207280). The remaining data are available within the article and its supplementary data files.

## Abbreviations

CRC: Colorectal cancer
DEG: Differentially expressed gene
dMMR: Deficient DNA mismatch repair
FDA: Food and Drug Administration
GFP: Green fluorescent protein
HTS: High-throughput screening
ICD: Immunogenic cell death
ICI: Immune checkpoint inhibitor
KLF: krüppel-like factor
MSI-H: Microsatellite instability-high
PBMC: Peripheral blood mononuclear cell
SREBF2: Sterol regulatory element-binding transcription factor 2
TIME: Tumor immune microenvironment
