# Supplementary material for: Phenotypic screening platform identifies statins as enhancers of immune cell-induced cancer cell death
Source: BMC Cancer. 2023 Feb 17;23:164. doi: 10.1186/s12885-023-10645-4 (PMC9938546; doi:10.1186/s12885-023-10645-4)
Supplement: Supplementary file 5 — Supplementary Material 5 [file 12885_2023_10645_MOESM5_ESM.docx]

Supplementary Figure S1

**Figure S1. Assay quality assessment and example images. (a)** GFP signal quantified in HCT116-GFP monoculture, PBMC monoculture, and co-culture every fourth hour for 96 h. Results are shown as mean ± SD from one representative experiment, n=16 for monocultures and n=32 for the co-culture. **(b)** Example images from the screen at 72 h. The images were obtained using the IncuCyte S3 and are presented without (upper panel) and with (lower panel) the overlaying mask that was used to quantify GFP-expression.

Supplementary Figure S2

**Figure S2. CytoDiff characterization of PBMCs.** Graph showing subpopulations of immune cells, displayed as % of total PBMC population, obtained from analysing PBMCs from three different donors using a CytoDiff flow cytometric system.

Supplementary Figure S3

**Figure S3. Treatment effect of statins in mono- and co-cultures over time.** Viability of HCT116-GFP cells measured over time in monoculture (1:0) or co-culture (1:4) with PBMCs from three different donors, treated with DMSO vehicle (0.01%, 0.1%) or statins (1 μM, 10 μM) for a total of 72 h. Data is shown as mean ± SD (n=3).

Supplementary Figure S4

**Figure S4. Pitavastatin treatment increases the expression of genes associated with pro-inflammatory stimuli.** Average log2 expression of DEGs (fold change > 2) found in co-culture treated with 1 μM pitavastatin but not in either of the monocultures.
